# Professional Quality of Life in Nursing: The Role of Psychological Resources—A Cross-Sectional Study

**DOI:** 10.3390/nursrep15120434

**Published:** 2025-12-07

**Authors:** Lovorka Brajković, Dora Korać, Vanja Kopilaš

**Affiliations:** Department of Psychology, Faculty of Croatian Studies, University of Zagreb, Borongajska Cesta 83d, 10000 Zagreb, Croatia; lbrajkov1@fhs.unizg.hr (L.B.); vkopilas@fhs.unizg.hr (V.K.)

**Keywords:** professional quality of life, PTSD, coping strategies, resilience, nurses

## Abstract

**Background/Objectives**: Nurses and nursing technicians are essential providers of patient care but remain highly vulnerable due to the demands of their profession, which can profoundly affect their professional quality of life. Understanding the risk and protective factors underlying different aspects of professional quality of life is crucial for fostering healthcare professionals’ overall well-being and ensuring high-quality care for patients. The aim of this study was to explore the relationships between professional quality of life, work-related factors, PTSD symptomatology and individual resources, including resilience and coping strategies. **Methods**: This cross-sectional study included 119 nurses from various nursing departments. A questionnaire comprising sociodemographic and work-related variables and four validated instruments, Professional Quality of Life Scale-5 (ProQOL-5), PTSD Checklist for DSM-5 (PCL-5), Brief-COPE and Brief Resilience Scale, was used for data collection. **Results**: Findings revealed moderate to high compassion satisfaction among nurses and technicians, as well as low to moderate burnout and moderate levels of secondary traumatic stress. Compassion satisfaction was positively associated with problem-focused and emotion-focused coping, whereas higher levels of compassion fatigue (burnout and secondary traumatic stress) were associated with avoidant coping, greater PTSD symptom severity, and lower resilience. Resilience, problem-focused coping, and PTSD symptom severity were identified as significant predictors of professional quality of life. **Conclusions**: To support nurses’ and technicians’ well-being, healthcare organizations should encourage open conversations about the emotional demands of patient care and provide interventions that promote effective coping and address PTSD symptoms, ultimately helping to reduce compassion fatigue and enhance compassion satisfaction.

## 1. Introduction

Access to safe and adequate healthcare is an essential human right and represents a foundation not only of healthcare system but of society as a whole. At the same time, healthcare systems around the world are facing a number of challenges, including the rising demand for medical care due to demographic change, an aging population and the increasing prevalence of chronic diseases [1]. Healthcare professionals have a crucial role in meeting these demands and providing care. However, there is increasing concern about diminishing job satisfaction and quality of life among them, both of which are largely attributable to increasing workload, lack of resources and occupational stress [2].

Nurses and nursing technicians represent a particularly vulnerable group, as there is a noticeable shortage in this profession worldwide. As well as dealing with a wide range of tasks such as administering medication, assessing patients’ needs and responses to treatment and working with interdisciplinary healthcare teams, the nursing profession is also unique in its prolonged and emotionally intense contact with people in distress [3]. Compared to other healthcare professionals, nurses and nursing technicians are more frequently exposed to fear, suffering, pain, and death, with emotional support and compassion for patients and their families being a central aspect of their role [4].

The holistic nature of the nursing profession makes nurses and technicians invaluable in the eyes of patients, but the challenges they face can have a negative impact on their well-being, job satisfaction, and ultimately their ability to provide quality care. Within this context, Professional Quality of Life (ProQOL) provides a useful framework for understanding both the positive and negative consequences of caregiving roles [5]. The positive dimension of professional quality of life is compassion satisfaction (CS), which reflects the sense of fulfillment and purpose derived from assisting others and supporting individuals experiencing or recovering from trauma. In contrast, the negative dimension is captured by compassion fatigue (CF), characterized as the emotional and physical burden that arises from continuous exposure to patients’ suffering in highly demanding clinical settings [6,7].

It consists of two components: burnout and secondary traumatic stress (STS; [8]). Burnout refers to feelings of exhaustion, helplessness, and frustration, often accumulated over time and typically arising from excessive workloads and unsupportive work environments. STS reflects the potential consequences of being indirectly exposed to the stressful or traumatic experiences of those who they help [8]. Recent studies have shown that CF is significantly prevalent among nurses working in a variety of settings, including oncology, hospice and intensive care, obstetrics and gynecology [9,10,11,12,13]. Increased levels of burnout were observed in 22% to 62.79% of nurses, while STS was found in 22% to 43% of cases [14,15,16].

The concept of ProQOL has been closely linked to the subjective well-being and job satisfaction of healthcare professionals, with previous research emphasizing the substantial physical, psychological, and emotional consequences of CF, particularly among nurses [17,18,19]. It has been associated with symptoms such as headaches, difficulties in retaining information, irritability, and fatigue [20]. A high level of exhaustion and frustration resulting from work overload and exposure to traumatic situations in the workplace can further impair nurses’ capacity to deliver comprehensive patient care [18]. Emotional detachment and numbness, often associated with CF, can lead to negative attitudes toward professional roles, diminished empathy and emotional connection, and a routinized approach to caregiving, all of which may potentially compromise patient health outcomes [21]. At the organizational level, negative aspects of caring for patients have been associated with increased absenteeism, high staff turnover, a greater intention to leave the profession, and an overall workforce shortage in the healthcare sector [22,23,24].

Alongside the prevalence and potential adverse consequences of diminished professional quality of life, numerous studies have examined factors that contribute to CS and CF. Socio-demographic variables, such as age, gender, marital status, and educational level, have been found to be associated with CF [25,26]. Among the work-related factors most frequently associated with CF are years of nursing experience, the type of department, shift work, and the presence of a leadership role [15,27,28]. Younger or less experienced nurses tend to report lower levels of CS and are at greater risk of burnout. At the same time, prolonged exposure to stress and traumatic events appears to be more pronounced among nurses with longer work experience [15]. Additionally, night shifts, irregular working hours, and overtime have been identified as contributing factors to increased risk of CF [29].

Although variability in the prevalence of CF has been partly attributed to demographic (e.g., age, gender) and work-related factors (e.g., years of experience, worked hours per day, professional role), findings regarding their association with ProQOL remain somewhat inconsistent [7,9,24]. Consequently, numerous studies emphasize the need to examine modifiable factors, with particular attention to individuals’ psychological resources [21].

Among these factors, resilience is widely recognized as an important protective resource for maintaining nurses’ well-being, psychological functioning, and job satisfaction, thus contributing to improved quality of patient care [30,31]. It enables individuals to recover from traumatic experiences and to adapt flexibly to the shifting demands of stressful environments—an ability that is especially crucial within the context of healthcare [32]. Resilience can be viewed as a mediator between exposure to acute traumatic events or chronic stress, such as that experienced in the nursing profession, and various psychological, physical, and behavioral outcomes [33]. Previous studies have confirmed the complex interplay between resilience and professional quality of life, showing that nurses with higher resilience report greater CS and lower CF [34,35]. Resilience promotes a greater sense of fulfillment and satisfaction in nursing care, which in turn is associated with lower levels of perceived burnout and STS [36]. Furthermore, resilience has been identified as a significant mediator in the relationship between prior exposure to traumatic events and both CS and CF [35]. Individuals with higher resilience cope more effectively with workplace stress and negative emotions, enabling nurses to mitigate emotional exhaustion and burnout and better manage the consequences of indirect exposure to patients’ stressful or traumatic experiences [37].

Coping is a dynamic concept that encompasses the cognitive and behavioral strategies individuals employ to manage internal or external demands perceived as challenging or exceeding their resources [38]. Nurses report using a variety of problem-focused and emotion-focused coping strategies to manage stressful situations and reduce STS, including crying, planning activities with loved ones, and spending time with family and friends [21]. In particular, avoidance has been shown to worsen professional quality of life by increasing CF, while decreasing CS [39]. Conversely, employing problem-focused coping strategies—such as finding positive aspects in difficulties—and seeking social support are associated with lower levels of burnout and STS [40].

Continuous exposure to the suffering and trauma of patients, particularly within high-intensity work environments, combined with potential exposure to stressful events in personal life, may exceed an individual’s coping resources and result in long-term psychological consequences. One such possible outcome is the presence of symptoms associated with post-traumatic stress disorder (PTSD), and in severe cases, the development of a formal PTSD diagnosis [41]. Previous studies have found a moderate to high prevalence of PTSD symptoms among nurses [42,43]. Interest in exploring the relationship between PTSD symptomatology and professional quality of life intensified during the COVID-19 pandemic when it was found that nurses who exhibited higher levels of PTSD and STS demonstrated lower CS and increased burnout [44]. However, this significant relationship between PTSD symptoms, CS and CF was also observed in the post-pandemic period [45].

Given the detrimental impact of compassion fatigue on both nurses’ well-being and the quality of care provided, as well as the protective role of compassion satisfaction, it is essential to investigate the risk and protective factors that shape these key dimensions of professional quality of life. The aim of this study is to examine the relationships between professional quality of life, selected work-related factors, PTSD symptomatology, and individual resources, namely resilience and coping strategies, in nurses and nursing technicians. In addition, the contribution of specific psychological risk and protective factors to the prediction of the different dimensions of ProQOL will be investigated.

## 2. Materials and Methods

### 2.1. Participants and Procedure

To examine the relationship between professional quality of life, psychological risk and protective factors, and specific aspects of the work environment at a single time point, a cross-sectional study design was employed.

The study was conducted at two major hospital institutions in the capital city: Clinical Hospital Centre Zagreb, the largest hospital in Croatia, providing comprehensive tertiary care across diverse medical specialties, and Clinical Hospital Dubrava, a key acute trauma and referral center. Including both public institutions ensured access to a diverse group of nurses and technicians with varying specialties and experience. Invitations to participate in the online survey were also distributed through selected local nursing associations. The inclusion criteria required participants to be registered nurses/technicians who had spent part of their careers in direct patient care and were actively employed at the time of completing the questionnaire. Data collection was conducted over a four-month period, from August to November 2024. At the beginning of the survey, participants were presented with an information sheet outlining the voluntary and anonymous nature of their participation, followed by an electronic consent form.

An a priori power analysis was conducted using G*Power version 3.1. Assuming a medium effect size of 0.15, a statistical power of 0.80, and a significance level of 0.05, the analysis indicated that a minimum of 92 participants would be required. The study initially included 119 nurses and nursing technicians from various departments, of whom 112 (94%) were female. The age range was between 19 and 73 years, with an average age of 42 years and 6 months (M = 42.46, SD = 13.8). Two participants (F = 1; M = 1) were excluded from further analyses because they were not actively employed (i.e., they were retired). The final sample comprised 117 nurses and technicians, with a mean age of 42 years (M = 41.97, SD = 13.37).

### 2.2. Instruments

Participants completed a structured survey, which included a sociodemographic questionnaire and validated measures for assessing professional quality of life, level of PTSD symptomatology, coping strategies and resilience.

Sociodemographic questionnaire: A structured questionnaire was used to gather sociodemographic information from participants, such as age, gender, marital/relationship status, children (Yes/No), education level and employment status. Occupational-related questions included years of professional experience, area of practice, number of overtime hours worked, working in shifts (Yes/No), and whether the participant held a supervisory position (Yes/No).

Professional Quality of Life Scale–Version 5 (ProQOL-5 [8]). The ProQOL-5 is a validated instrument that reflects quality a person feels in relation to one’s work as a helper. The questionnaire consists of 30 items divided into three subscales: compassion satisfaction, and burnout and secondary traumatic stress that reflect compassion fatigue. Each subscale comprised 10 items in which respondents rated the frequency of specific emotions or events experienced over the past 30 days on a 5-point Likert scale ranging from 1 (never) to 5 (very often). The total score is calculated by summing up estimates on each item, and it ranged from 10 to 50 for each subscale, with scores categorized into low (22 or below), average (23–41), or high levels (42 and above). A higher score on compassion satisfaction (CS) subscale indicated greater satisfaction derived from the ability to provide effective care, support others, and contribute positively to the work environment or society. A higher score on Burnout reflected higher presence of exhaustion, frustration, and hopelessness related to work, while higher score on secondary traumatic stress (STS) subscale indicated work related, secondary exposure to stressful events or traumatic experiences.

Previous Cronbach’s alpha values indicate good internal reliability for these subscales, with values above α = 0.83 for CS, above α = 0.82 for Burnout, and above α = 0.90 for secondary traumatic stress subscale [46,47]. The Cronbach’s α for ProQOL subscales in this study were as follows: 0.89 for the overall scale, 0.92 for the CS subscale, 0.78 for the Burnout subscale and 0.87 for the STS subscale.

PTSD Checklist for DSM-5 (PCL–5 [48]). PCL-5 is a self-reported instrument used to assess the presence of posttraumatic stress disorder (PTSD). It consists of 20 items that reflect 20 symptoms of PTSD. Participants rated the degree to which each symptom was present in the past month using a 5-point scale (0—Not at all; 4—Extremely). Total score can range from 0 to 80, with higher scores indicating more severe symptoms, and scores of 33 or above indicating significant PTSD symptoms that interfere with daily functioning [49]. In addition to the total score, results can be examined across four subscales, which correspond to the PTSD symptom clusters defined in the DSM-5: re-experiencing, avoidance, negative alterations in cognition and mood, and hyperarousal.

The scale shows good internal consistency in previous studies with a Cronbach alpha ranging from 0.83 to 0.97 ([50,51]. Internal consistency in this study was α = 0.97.

Brief Coping Orientation to Problems Experienced Inventory (Brief-COPE [52]). Brief-COPE was used to assess effective and ineffective ways to cope with stressful life events. It consists of 28 items grouped into three subscales that reflect different coping styles: Problem-focused, Emotion-focused, and Avoidant coping. Participants rated the statements on a 4-point scale, ranging from 1—“I haven’t been doing this at all” to 4—“I’ve been doing this a lot”. Subscale scores are calculated as the mean of item responses, with higher scores indicating a greater tendency toward a particular coping style. Previous Cronbach’s alpha values (α = 0.90) indicate excellent internal consistency [53]. In the present study, the values of Cronbach α were 0.84 for the Problem- focused coping, 0.78 for Emotion-focused coping, and 0.78 for Avoidant coping.

Brief Resilience Scale [54]. Brief Resilience Scale is a six-item measure of resilience. Participants assessed items on a 5-degree Likert type scale, ranging from 1 (I completely disagree), to 5 (I completely agree). The total score represents the average value on all items, with higher scores reflecting a higher degree of resilience. Previous Cronbach’s alpha values (α = 0.93) indicate excellent internal reliability [55]. The Cronbach alpha for the current study indicates good reliability of the Brief Resilience Scale (α = 0.82).

### 2.3. Data Analysis

The dataset was analyzed using the IBM SPSS Statistics program (version 26). Prior to conducting the statistical analysis, it was first examined for outliers. The values of all participants were below three or more standard deviations from the mean on the observed variables indicating the absence of outliers. Additionally, all participants provided responses to every item included in the questionnaire, resulting in a complete dataset with no missing data. Significance was set at *p* < 0.05.

Descriptive analyses were conducted to compute means and standard deviation of continuous variables, as well as the frequencies of categorical variables. Considering certain variables had asymmetric distributions, both the mean and median values have been reported.

Given the sample size and the absence of outliers [56], a Pearson correlation analysis was performed to examine the relationship between dimensions of professional quality of life and certain work characteristics, PTSD symptomatology, coping strategies and resilience.

To examine the contribution of PTSD symptomatology, coping strategies and resilience in explaining professional quality of life, three hierarchical regression analysis were conducted. To determine whether hierarchical regression analysis was suitable for our data, indicators of multicollinearity and Mahalanobis and Cook distances were calculated.

## 3. Results

### 3.1. Sociodemographic Characteristics

The majority of observed nurses and technicians had master’s degree in nursing (41.9%), were married (65.8%) and had children (73.5%). Regarding work experience, the study included experienced nurses/ technicians, with nearly 70% reporting more than 10 years of professional experience, and with average length of working experience of 20 years (M = 19.9, SD = 13.67). Participants were almost evenly distributed between institutions providing primary and secondary healthcare services, The majority (82.9%) did not occupy supervisory positions, and did not work in shifts (53.8%). However, approximately half of the respondents (50.4%) reported working overtime in the month preceding their participation in the study, with an average of nearly 18 h of overtime work (M = 17.8, SD = 13.2). Additional sample characteristics are presented in Table 1.

### 3.2. Descriptive Statistics

Table 2 shows descriptive data for the dimensions of professional quality of life, PTSD symptomatology, coping strategies and resilience.

In terms of professional quality of life, the nurses and nursing technicians included in this study reported moderate to high levels of CS, i.e., the positive feelings derived from being able to help others through their work and to do it effectively (Table 2). According to the established categorization, 52.1% of participants fell into the moderate CS category, while 46% were classified as having high compassion satisfaction. The average scores on the subscales reflecting CF indicate low to moderate levels of burnout, and a moderate level of distress related to exposure to secondary stressful or traumatic experiences in the workplace. Nearly half of the participants (47.9%) demonstrated low burnout, while over half (56.4%) reported moderate levels of STS. Participants reported an average sum score of 32.12 (SD =  21.32) on the PCL-5 with almost half of the sample (49.6%) met or exceeded a recommended cut score of 33 for identifying a probable PTSD diagnosis. When considering the symptom clusters separately, nurses and technicians in this study experienced mild hyperarousal, reflected in hypervigilance and sleep difficulties, together with heightened negative emotions such as fear, anger, and guilt. A greater tendency was also observed toward avoidance of trauma-related thoughts and emotions, along with a moderate frequency of re-experiencing the traumatic event(s) through intrusive thoughts and distressing memories. With regard to coping strategies, participants most frequently employed problem-focused coping, while avoidant coping was the least commonly reported. Additionally, they demonstrated a moderate tendency toward emotion-focused coping and a slightly above-average level of resilience.

### 3.3. Correlation Analysis

To determine the association between ProQOL, PTSD symptom severity, coping strategies, resilience and certain work characteristics, Pearson’s correlation analysis was conducted (Table 3).

Nurses and nursing technicians who reported higher levels of compassion CS tended to score lower on the burnout subscale. In contrast, those who reported greater exposure to work-related STS exhibited higher levels of burnout. Significant correlations were found between certain aspects of professional quality of life and coping strategies. Greater CS was associated with a higher tendency to engage in problem- focused and emotion- focused coping. Participants with higher levels of CF (as indicated by increased scores on Burnout and STS), were more likely to use avoidant coping strategies. Additionally, significant, but weak, positive correlations were established between STS and problem- focused and emotion-focused coping. Moderate, positive correlations were observed between PCL-5 scores and compassion fatigue, suggesting that nurses and nursing technicians with greater PTSD symptom severity (PCL-5) tend to experience higher levels of frustration, anger, and hopelessness related to their work, as well as increased secondary exposure to stressful and traumatic events. PTSD symptom severity showed positive correlations with all three coping strategies, with the strongest association observed for avoidant coping. Higher resilience was associated with fewer PTSD symptoms, and lower level of burnout and STS. With regard to work-related characteristics, participants with longer professional experience reported higher levels of STS, whereas those not engaged in shift work reported greater CS and more frequent use of problem-focused coping strategies.

### 3.4. Regression Analysis

Three hierarchical regression analysis was used to determine the factors contributing to compassion satisfaction and compassion fatigue (burnout and secondary traumatic stress. Prior to conducting these analyses on our data, multicollinearity indicators, i.e., Variance Inflation Factor (VIF) and Tolerance, were observed. Considering the values of these indicators did not exceed critical thresholds (VIF < 10; Tolerance > 0.2), no violations of the multicollinearity assumption were identified. Furthermore, Cook’s distance values below 1 and Mahalanobis distance values under 25 suggest the absence of multivariate outliers in the analyzed data [56]. The first block in all three hierarchical regression analysis included PTSD symptomatology, the second block contained resilience and the third block included coping strategies (Table 4).

The performed regression analysis with CS as the outcome variable showed the statistical significance of second and third block, i.e., after coping strategies entered the model (FModel1 = 0.939; *p* > 0.05; FModel2 = 5.649; *p* < 0.001; FModel3 = 4.670; *p* < 0.001). All predictors included in the analysis explained 17.4% (R^2^adj = 13.7%; *p* < 0.001) of the variance in compassion satisfaction, However, only problem-focused coping strategies proved to be significant predictor (Table 4).

In the second hierarchical regression analysis, burnout was predicted using the same set of predictors (Table 4), accounting for a total of 36.1% of the variance (R^2^adj = 33.2%; *p* < 0.001). All three blocks were statistically significant (FModel1 = 41.51; *p* < 0.001; FModel2 = 13.98; *p* < 0.001; FModel3 = 12.52; *p* < 0.001). The first block (PTSD symptomatology) explained 26.5% of the variance (R^2^adj = 25.9%; *p* < 0.001), the second block contributed an additional 6.8% (R^2^adj = 30.9%; *p* < 0.001), while the inclusion of resilience in the final model contributed an additional 2.8% (R^2^adj = 33.2%; *p* < 0.05). The significant predictors were the severity of PTSD symptoms and resilience.

The results of the hierarchical regression analysis, with STS as the outcome variable, demonstrated that all three blocks of predictors were significant (FModel1 = 83.2, *p* < 0.001; FModel2 = 22.28, *p* < 0.001; FModel3 = 21.02, *p* < 0.001). All predictors included in the analysis explained 48.6% (Radj= 46.3%; *p* < 0.001) of the variance in STS, with the first block explaining 42% of the variance (Radj = 41.5%; *p* < 0.001). PTSD symptomatology and resilience were identified as significant predictors of secondary exposure to stressful events or traumatic experiences.

## 4. Discussion

Compassion satisfaction and compassion fatigue constitute central components of healthcare professionals’ overall well-being—both mental and physical—and, as such, play a crucial role in determining the quality of care delivered. The present study aimed to examine the professional quality of life of nurses and nursing technicians, as well as its’ relationship with specific psychological risk and protective factors.

When observing specific dimensions of ProQOL, a relatively high level of CS was found, alongside a low level of burnout and moderate STS. These findings suggest that, while nurses and technicians generally derive considerable satisfaction from their caregiving roles, they also report moderate impairment in functioning as a result of exposure to the stressful and traumatic experiences of the individuals in their care. In comparison with previous studies, our results indicate somewhat higher levels of CS and relatively lower levels of burnout [5,13,25]. High levels of CS, accompanied by low to moderate burnout and the presence of STS, were also reported in a recent study conducted among Croatian nurses working in different hospital departments [46]. Similar patterns in the dimensions of ProQOL were established in study with oncology nurses [16]. Discrepancy in the levels of CS and CF may be attributable to differences in work environments (organizational support, work departments), as well as sample characteristics, such as age, experience, and field of work. It is worth noting that our sample predominantly consisted of relatively experienced nurses/technicians, most with over 10 years of practice, the majority of whom were employed as health visitors for newborns and their parents, as well as in oncology wards. In addition, the timing of data collection should also be considered, as studies conducted immediately after the COVID-19 pandemic reported higher levels of CF [57]. When examining the relationships among specific dimensions of ProQOL, the fulfillment derived from helping others (CS) was found to be associated with the lower level of burnout, but not with the presence of STS. Furthermore, nurses and nursing technicians who experienced higher levels of burnout also reported higher STS, which has been confirmed in previous studies conducted with nurses from different clinical settings.

Unlike previous research, which has demonstrated a significant association between years of nursing experience and all dimensions of ProQOL, the present study revealed a significant relationship only with STS [57,58]. More experienced nurses and technicians who have been in the profession for longer reported more frequent secondary exposure to stressful events or traumatic experiences stemming from contact with patients. However, a recent study on oncology nurses found no associations between years employed as a nurse and levels of BO and STS [59]. In addition, a significant association was observed with overtime work, such that nurses and nursing technicians who worked beyond the standard schedule reported lower levels of CS. No significant associations were found with either burnout or STS. Similarly, no relationship emerged with shift work, which is partly in contrast to earlier studies that reported significant association with at least one dimension of ProQOL (with CS [59] and STS [60]). The observed lack of significant associations between specific work-related factors and ProQOL is consistent with recent research conducted among nurses in various clinical settings [5,61].

Almost 50% of the nurses and technicians in the present study had scores suggestive of a possible PTSD diagnosis. This proportion is somewhat higher than in prior studies, which may be partly explained by differences in the cut-off scores used to determine probable PTSD [62]. Participants reporting higher levels of PTSD symptomatology also exhibited higher levels of compassion fatigue, which was a significant predictor of both burnout and STS. The significant relationship between PTSD and components of ProQOL is consistent with previous research [44]. One possible explanation for the association with CF is that prior exposure to traumatic events and their functional consequences may hinder nurses’ ability to adapt to stressful environments, leading to the adoption of maladaptive coping strategies and, ultimately, greater vulnerability to burnout and STS [63]. This is also supported by the significant associations found in this study between higher PTSD symptoms, lower resilience, and a greater tendency to use avoidant coping strategies.

Examining the relationship between resilience and ProQOL dimensions, it was evident that nurses and technicians with higher levels of resilience exhibited lower levels of burnout and fewer symptoms of STS. Furthermore, resilience emerged as a significant predictor of burnout and secondary exposure to stressful and traumatic events. The observed significant relationship between resilience and CF has been confirmed among nurses/technicians working in various clinical departments, including mental health nurses, oncology nurses, and intensive care unit nurses [36,60,64].

In this study, nurses and nursing technicians most frequently reported using problem-focused coping strategies, while avoidance strategies were least frequently used [63,65] which is confirmed in this study. Participants who more frequently employed problem-focused and emotion-focused coping reported higher levels of CS, but also greater presence of STS. One possible explanation for the positive association between emotion-focused coping and STS lies in heterogeneous nature of emotion-focused coping, which includes both adaptive strategies, such as seeking emotional support, and maladaptive strategies, such as self-blame. Although the observed association between problem-focused coping and elevated STS was small, it may be explained by the nature of the stressors encountered. In environments with persistent and only partially controllable demands, individuals who typically rely on problem-focused strategies may be unable to apply them effectively. The continuous emergence of new stressors, combined with limited time and resources, can further impede adaptive coping and contribute to higher STS. A significant positive association between compassion fatigue and problem-focused coping has been reported among nurses in various departments [40]. Similarly, no protective effect of adaptive strategies such as acceptance and active coping was identified [66]. Consistent with previous research, avoidance coping was found to worsen professional quality of life, by increasing burnout and STS [21,67]. Among coping strategies, only problem-focused coping was found to be a significant predictor of CS. Recent studies have also shown that adaptive coping mechanisms, such as problem-solving and emotion-focused strategies in terms of emotional support or religious coping, improve professional quality of life [21,68].

There are several limitations of this study that should be considered when interpreting the results. Due to the cross-sectional design, it was not possible to establish causal relationships between professional quality of life and the observed psychological variables. In addition, the possibility of response bias cannot be ruled out, as nurses and technicians who were relatively satisfied with various aspects of their profession or had stronger coping resources and resilience were more likely to participate in the study, while those who were more exposed to traumatic or stressful events and were “burned out” were not willing to participate. Results relied on a convenience sample of only 117 nurses/technicians, which may reduce the generalizability of the findings.

Future research should include more diverse samples (e.g., younger nurses) and ensure a more balanced representation across different departments and healthcare settings. Although this study included nurses and nursing technicians from various units, their distribution was uneven, which may limit the representativeness of the sample.

The study also examined a narrow range of work-related variables and did not include characteristics of the work environment, which may vary across hospital departments. To gain a deeper understanding of ProQOL among nurses, greater emphasis should be placed on organizational factors such as workload, working conditions, interpersonal relationships, and leadership. While the focus was primarily on individual resources, family-related factors were not considered, despite the importance of work–life balance in shaping well-being. Qualitative approaches may also provide a deeper understanding of factors underlying high compassion satisfaction and specific adaptive coping strategies used in stressful situations.

## 5. Conclusions

This study underscores the importance of examining nurses’ and nursing technicians’ professional quality of life alongside its complex interplay with individual psychological resources, especially problem-focused coping, resilience and the prevalence of PTSD symptoms. To mitigate burnout and functional difficulties associated with secondary traumatic stress, and to foster compassion satisfaction, healthcare organizations should promote open dialog about the emotional burden of patient care and implement interventions that support effective coping and address PTSD symptoms resulting from prior traumatic experiences. Additionally, interventions that raise awareness of compassion fatigue and educate nurses and technicians about associated risks and healthy work–life balance, combined with fostering a supportive culture of compassion, are essential. Although participants in this study demonstrated a generally favorable professional quality of life, with moderate to high compassion satisfaction and moderate levels of compassion fatigue, the findings underscore the need to further strengthen mental health support.

## Figures and Tables

**Table 1:** Sample characteristics (*N* = 117).

		f (%)
Education status	High school	34 (29.0)
	Bachelor’s degree	29 (24.8)
	Master’s degree	49 (41.9)
	PhD	5 (4.3)
Relationship status	Married	77 (65.8)
	Co-habiting/unmarried	12 (10.3)
	Divorced	8 (6.8)
	Widow	4 (3.4)
	Single	16 (13.7)
Field of work	Primary care	38 (32.5)
	Secondary care	39 (33.3)
	Tertiary care	29 (24.8)
	Education	8 (6.8)
	Other (e.g., sale representative)	3 (2.6)
Department	Home visitors (newborns)	23 (19.7)
	Internal medicine	16 (13.7)
	Oncology	15 (12.8)
	Surgery	11 (9.4)
	Intensive care	8 (6.8)
	Community healthcare	6 (5.1)
	Education	6 (5.1)
	Psychiatry/psychology	5 (4.3)
	Pediatrics	4 (3.4)
	Family medicine	4 (3.4)
	Emergency medicine	2 (1.7)
	Urology	2 (1.7)
	Gynecology	2 (1.7)
	Clinics	2 (1.7)
	Neurology	1 (0.8)
	Anesthesiology	1 (0.8)
	Other	9 (7.7)

**Table 2:** Descriptive data for the observed variables (*N* = 117).

	M (SD)	C (Min–Max)	Skew	Kurt	S-W
ProQOL-CS	39.73 (6.82)	40 (18–50)	−0.79	0.43	0.94 *
ProQOL-Burnout	23.27 (5.99)	23 (11–37)	0.22	−0.65	0.98
ProQOL-STS	25.43 (7.29)	24 (12–47)	0.68	0.32	0.96 *
PCL-5_total	32.12 (21.32)	32 (0–80)	0.25	−0.91	0.96 *
PCL-5_Re-experiencing	8.67 (6.03)	8 (0–20)	0.18	−1.22	0.94 *
PCL-5_Avoidance	3.69 (2.76)	4 (0–8)	0.04	−1.22	0.91 *
PCL-5_NegAlterations	10.37 (8.18)	8 (0–28)	0.48	−0.87	0.93 *
PCL-5_Hyperarousal	9.39 (6.42)	9 (0–24)	0.28	−0.67	0.96 *
Problem-focused	2.95 (0.63)	3 (1.25–4)	−0.52	0.14	0.96 *
Emotion-focused	2.56 (0.51)	2.58 (1.08–4)	−0.12	0.65	0.99
Avoidant coping	1.96 (0.55)	1.75 (1–4)	1.06	1.44	0.93 *
Resilience	3.25 (0.72)	3.17 (1.33–5)	0.05	0.27	0.98

* *p* < 0.05; M, mean; SD, standard deviation; C, median; Skew, skewness; Kurt, kurtosis, S-W, Shapiro–Wilk statistics; CS, compassion satisfaction; STS, secondary traumatic stress; PCL-5, PTSD Checklist for DSM-5; NegAlterations, Negative Alterations in Cognition and Mood.

**Table 3:** Correlation analysis of the observed variables (N = 117).

	1	2	3	4	5	6	7	8	9	10	11
ProQOL-CS (1)	-										
ProQOL-Burnout (2)	−0.57 **										
ProQOL-STS (3)	0.01	0.62 **									
PCL-5 (4)	−0.09	0.52 **	0.65 **								
Problem_focused (5)	0.35 **	−0.06	0.26 **	0.25 **							
Emotion_focused (6)	0.23 *	0.08	0.35 **	0.32 **	0.76 **						
Avoidant_coping (7)	−0.09	0.44 **	0.50 **	0.65 **	0.30 **	0.51 **					
Resilience (8)	0.17	−0.42 **	−0.46 **	−0.43 **	−0.01	−0.04	−0.41 **				
Experience (9)	−0.04	0.07	0.20*	0.16	0.05	0.06	0.18	−0.19 *			
Shifts (10)	0.05	−0.16	−0.02	−0.07	0.19 *	0.19 *	0.03	0.03	0.20 *		
Overtime (11)	0.23 *	−0.17	−0.08	−0.08	0.24 *	0.15	−0.11	0.01	0.03	0.37 **	-

* *p* < 0.05; ** *p* < 0.01; CS, compassion satisfaction; STS, secondary traumatic stress; PCL-5, PTSD Checklist for DSM-5; NegAlterations, Negative Alterations in Cognition and Mood.

**Table 4:** Hierarchical regression model predicting compassion satisfaction, burnout, and secondary traumatic stress (N = 117).

	Model 1				Model 2				Model 3			
	*B*	ß	t	SE	B	ß	t	SE	B	ß	t	SE
Predictors of CS												
PCL-5	−0.029	−0.09	−0.969	0.03	−0.029	−0.092	−0.806	0.037	−0.021	−0.066	−0.562	0.038
Problem_focused					4.041	0.371	2.722 **	1.484	4.037	0.370	2.717 **	1.486
Emotion_focused					0.893	0.067	0.444	2.010	0.639	0.048	0.315	2.032
Avoidant_coping					−2.141	−0.172	−1.349	1.587	−1.777	−0.143	−1.084	1.640
Resilience									0.844	0.089	0.891	0.947
R^2^	0.008				0.168				0.174			
Adjusted R^2^	0.001				0.138				0.137			
ΔR^2^	0.008				0.16				0.006			
F Change	0.939				7.169 **				0.794			
Predictors of Burnout												
PCL-5	0.145	0.515	6.443 **	0.022	0.119	0.423	4.134 **	0.029	0.103	0.367	3.538 **	0.029
Problem_focused					−2.267	−0.237	−1.945	1.166	−2.259	−0.236	−1.970	1.147
Emotion_focused					0.062	0.005	0.039	1.579	0.544	0.046	0.347	1.568
Avoidant_coping					2.515	0.230	2.018 *	1.246	1.825	0.167	1.442	1.265
Resilience									−1.601	−0.192	−2.191 *	0.731
R^2^	0.265				0.333				0.361			
Adjusted R^2^	0.259				0.309				0.332			
ΔR^2^	0.265				0.068				0.028			
F Change	41.51 **				3.79 *				4.8 *			
Predictors of STS												
PCL-5	0.222	0.648	9.121 **	0.024	0.194	0.567	6.066 **	0.032	0.170	0.497	5.346 **	0.032
Problem_focused					−0.016	−0.001	−0.013	1.298	−0.004	0.001	−0.003	1.252
Emotion_focused					1.962	0.138	1.116	1.758	2.696	0.189	1.574	1.713
Avoidant_coping					0.763	0.057	0.550	1.387	−0.286	−0.021	−0.207	1.382
Resilience									−2.436	−0.240	−3.054 **	0.798
R^2^	0.420				0.443				0.486			
Adjusted R^2^	0.415				0.423				0.463			
ΔR^2^	0.420				0.023				0.043			
F Change	83.199 **				1.568				9.325 **			

* *p* < 0.05; ** *p* < 0.01; B, unstandardized regression coefficient; β, standardized regression coefficient; t, t-statistic; SE, standard error; R^2^, coefficient of determination; Adjusted R^2^, adjusted coefficient of determination; ΔR^2^, change in coefficient of determination; F Change, F statistic for model change; CS, compassion satisfaction; STS, secondary traumatic stress; PCL-5, PTSD Checklist for DSM-5.

## Data Availability

The data presented in this study are available on request from the corresponding author.

## Public Involvement Statement

No public involvement in any aspect of this research.

## Guidelines and Standards Statement

This manuscript was drafted against the (STROBE) for a cross-sectional study, descriptive research.
